# Increased chromium and molybdenum blood levels after minimally-invasive repair of pectus excavatum

**DOI:** 10.1007/s00383-026-06303-y

**Published:** 2026-02-09

**Authors:** Michele Torre, Luca Genova Gaia, Federica Lena, Maria Grazia Calevo, Maria Raso, Sebastiano Barco, Francesca Di Gaudio, Giuliana Cangemi

**Affiliations:** 1 Pediatric Thoracic and Airway Surgery Unit, IRCCS Istituto Giannina Gaslini, Genova, Italy; 2 Department of Pediatric Surgery, IRCCS Istituto Giannina Gaslini, Via Gerolamo Gaslini, 5, Genova, 16147 Italy; 3 University of Genoa, DINOGMI, Genova, Italy; 4 Epidemiology and Biostatistics Unit, IRCCS Istituto Giannina Gaslini, Genova, Italy; 5 Chromatography and Mass Spectrometry Section, Department PROMISE, Quality Control and Chemical Risk (CQRC), University of Palermo, Palermo, Italy; 6 Biochemistry, Pharmacology and Newborn Screening Unit, Central Laboratory of Analyses, IRCCS Istituto Giannina Gaslini, Genova, Italy

**Keywords:** Thoracic surgery, Pediatric surgery, Pectus excavatum, Minimally invasive surgery, Blood metal levels

## Abstract

**Objectives:**

Minimally Invasive Repair of Pectus Excavatum (MIRPE) is the most popular technique to repair pectus excavatum in young patients. One or more metallic bars are inserted and maintained for at least 2–3 years. Only a few reports on possible metal release in these patients have been published. The study aimed to search for an increase in blood metal levels in patients after MIRPE and to investigate if surgical details (number of bars and stabilizers) were correlated with metal release.

**Methods:**

We have prospectively studied blood levels of chromium, nickel, molybdenum and manganese before bar implant in a group of patients undergoing MIRPE between 2017 and 2019 and, in the same patients, at the moment of bar removals between 2020 and 2022. All our patients had the same stainless-steel bar. Blood samples were analysed using inductively coupled plasma mass spectrometry.

**Main results:**

We included a total of 53 (10 females) patients. Median age at MIRPE was 15.4 years. After a median bar maintenance time of 3.1 years, we observed significantly higher mean levels of chromium (2.43 vs. 0.52 µg/L) and molybdenum (1.87 vs. 0.35 µg/L; *p* < 0.05). Nickel (4.24 vs. 80.80 µg/L) and manganese (12.69 vs. 19.81 µg/L) were also higher, although not statistically significant. No differences were found regarding the number of bars, stabilizers implanted or gender. No patients had clinical symptoms of metallosis.

**Conclusions:**

We demonstrated that metal blood levels increase in patients with retrosternal bars after MIRPE. Clinical implications of our finding are still unknown.

## Introduction

Minimally Invasive Repair of Pectus Excavatum (MIRPE), also known as the Nuss procedure, involves the placement of one or more metal bars inside the chest to correct a congenital deformity called pectus excavatum, where the chest appears sunken. The metal bar used in MIRPE is typically made of surgical stainless steel or titanium. Several reports have demonstrated the effectiveness and safety of MIRPE, with overall excellent short and long-term outcomes [1, 2] and immediate improvement in chest wall morphology. The procedure requires the metal bars to be secured to the rib cage and between themselves and one or more metal stabilizers are usually put in place for this purpose. Bars and stabilizers are typically removed after three years from the positioning, when the chest wall is permanently remodelled.

Despite their temporariness, metal devices could be responsible for the development of side effects such as irritative symptoms. Elevated trace metal levels, in particular nickel and chromium, have been found after MIRPE at bar removal [3, 4]. Furthermore, Fortmann et al. [5] pointed out that elevated blood nickel levels can persist for 30 months after the bar removal.

Several studies report erosion and dissemination of the components of metallic prosthesis in the body in orthopedic surgery; however clinical consequences remain unclear [6, 7]. In general, the toxic action of metals is correlated to the oxidative stress of the subject and the damage level is difficult to predict; therefore, increased trace metals levels could present with a wide variety of signs and symptoms, from the absence of symptoms to local effects to systemic consequences. Furthermore, it should be noted that studies are limited by technical challenges in analyzing trace metal levels by inductively coupled plasma mass spectrometry (ICP-MS), a technique that is not commonly available in clinical laboratories.

Considering the high incidence of pectus excavatum [8] and the popularity of MIRPE, increasing the available data on possible complications and side effects is crucial for improving patient outcomes and safety.

The aim of this prospective, longitudinal, observational cohort study was to assess any differences in blood trace metal levels as a consequence of MIRPE by measuring metals in patients’ blood longitudinally at the time of implantation and at the time of bar removal.

## Materials and methods

The study included 54 patients undergoing MIRPE between 2017 and 2020 and consequent bar removal procedures between 2020 and 2022 at Giannina Gaslini Institute, a pediatric tertiary care institution.

The study was approved by the institutional Internal Review Board (IRB protocol number 0015221/20) and received ethical approval (protocol number MT/IGG01/2015). For each surgical procedure, a detailed written and oral informed consent was obtained by the patient or by the person or people holding parental rights.

All patients received the same implanted metal devices that were all stainless steel, manufactured by Intrauma (Intrauma, Torino, Italy). Chemical composition (ISO 5832-1:2016) is reported in Table 1. Bars and stabilizers are made of the same composition.

Allergies to metals or other substances were investigated in patients and in their families. In patients with a positive history of metal reactions a different type of device was chosen.

Exclusion criteria included: differences in chemical composition of the bar, contamination of the blood sample, absence of follow up after MIRPE, patient with a positive history of metals allergy or adverse reaction, patients with previous surgical implantation of metallic synthesis devices.

MIRPE was performed via right thoracoscopy. According to patient characteristics and age, one or more bars were inserted. In order to stabilize the bar, one or more metal stabilizers were used.

Follow up was regularly scheduled at our Institute. For each patient, blood metals were evaluated before the implant and at bar removal. In particular, before starting each surgical procedure and after general anaesthesia induction, a blood sample was obtained via peripheral venous access using a syringe without a needle and then collected in metal-free tubes (Vacuette Greiner Bio-One International) after discarding the first 3 ml, to limit any possible contamination.

Blood samples were stored at -30 °C until they were shipped to the reference laboratory (Haematology and Rare Disease Department, United Hospitals Villa Sofia-Cervello Hospital, Palermo, Italy) for analysis. Metal quantification was performed by inductively coupled plasma mass spectrometry (ICP-MS -Thermo Scientific iCAP TQe) equipped with a collision cell. Using a minor modification of the previously published method [3]. All chemicals used for the ICP-MS analysis were of ultrapure grade. Nitric Acid 65% w/w and hydrogen peroxide 30% were purchased from J.T.Baker. Ultra-pure water (resistivity of 18.2 MΏ) was obtained by Nano-pure Diamont-Bearnstead. Working standard solutions for each element were prepared daily using high-density polyethylene bottles by stepwise dilution of the multi-element stock standard solution (1000 ± 5 g mL^− 1^) (DBH, Merck or CPI International) in a solution of HNO_3_ 1%. Blood samples were digested and mineralized using a microwave oven (CEM, Italy) with a high-pressure rotor. A 2 ml aliquot of blood was transferred into quartz vessels and treated with 3 mL of HNO_3_ (69%), 2 mL of H_2_O_2_ (30%) and 100 µL of a solution containing Au at 100 ng/L. The instrumental conditions used for the microwave digestion were: 2 min at 100 °C, *P* = 500 PSI, 1 min at 150 °C, *P* = 500 PSI and 5 min at 200 °C *P* = 500 PSI. Optimization was performed by using a 1 µg L^− 1^ solution of ^7^Li, ^59^Co, ^115^In, ^140^Ce and ^238^U (in HNO_3_ 1%). Moreover, the ^140^Ce^16^O^+^/^140^Ce^+^ ratio was used to check the level of oxide ions in the plasma and instrumental parameters such as RF power and carrier gas flow were optimized and the level of doubly charged ion was monitored by means of the signal Ba^2+/^Ba^+^. In order to minimize polyatomic interferences, such as the chromium signal at m/z 53 that is affected by ^40^Ar^13^C^+^ and ^37^Cl^16^O^+^, or the nickel signal at m/z 58 that is affected by ^42^Ca^16^O^+^. A collision cell was used. The formation of interfering polyatomic species was monitored by evaluating the ratios CeO/Ce and Ba^2+^/Ba^+^ maintained less than 3% by setting the voltages applied to the instrument lenses. The instrumental parameters used are listed in Table 1. A solution of HNO_3_ 1 M was run during the analysis to guarantee a negligible memory effect. Blank samples were also analysed and subtracted from each determination. Each solution was measured in triplicate and ICP-MS analyses were carried out with a classical external calibration approach from 0.05 to 500 ng/ mL for each investigated element using 89Y (50 ng/ mL) as an internal standard to compensate for any signal instability; all data in cps were normalised to the internal standard. The operating conditions are shown in Table 2.

Descriptive statistics were generated. Data were expressed as mean and standard deviation, as median and range for continuous variables and as absolute or relative frequencies for categorical variables. Data distribution was analysed using the Kolmogorov-Smirnov test and non-parametric statistics were considered.

The paired Wilcoxon test was used to compare the trace metal levels present before inserting the bars and when removing the bars. Differences between groups were evaluated using the Mann-Whitney test for continuous variables and χ2 or Fisher’s exact test for categorical variables. The Spearman correlation analysis was used to observe the correlations between the interval time from bar placement to removal and the increase of the trace metal levels.

A P-value < 0.05 was considered statistically significant; all P values were based on two-tailed tests. Statistical analyses were performed using SPSS (SPSS Inc, Chicago, Il, USA).

The reference values for blood metal thresholds provided by the Italian National Institute of Health (Istituto Superiore di Sanità) were applied [9].

## Results

Fifty-four patients were enrolled: 46 boys (85.2%) and 8 girls (14.8%). Mean age was 14.9 years (SD ± 3.82) at first procedure and 18.3 years (SD ± 3.27) at bar removal. The median time elapsed from bar placement to removal was 3.16 years (range 2.15–3.81 years). One patient was excluded from the analysis because of a very high Nickel value (> 10000 µg/l) recorded at the time of bar placement. The same patient had values slightly above the limit of normal at bar removal; therefore, the first sample was considered contaminated.

For each metal of interest, differences between the mean value of trace metal levels before MIRPE and at bar removal were calculated. Results are summarized in Table 3. Chromium and molybdenum mean values were significantly increased (from 0.52 ± 0.43 to 2.43 ± 9.57 µg/l; *p* < 0.001 and from 0.35 ± 0.39 to 1.87 ± 1.29 µg/l; *p* < 0.001, respectively), without any correlation with the interval time from placement to removal. Nickel and manganese mean values were also increased, but the differences were not statistically significant. For each metal of interest, the number of patients with values above the reference threshold before and after metal devices was compared. As regards chromium, the patients with values above the limits pre MIRPE were 47.2% while post MIRPE 62.3%, with no significant increase (*p* > 0.05). No significant differences were observed also for manganese, molybdenum and nickel.

Patients with more than one bar did not display a higher level of metals after MIRPE compared to those with one bar only (Table 4).

Considering the number of stabilizers, no significant differences were found between the number of stabilizers and the differences between blood levels.

No significant difference based on age at first procedure or time before the second procedure could be found.

A higher mean value of nickel among female patients could be observed, although the analysis of differences between male and female subjects didn`t reveal any statistically significant difference.

## Discussion

In recent years, interest in studying the correlation between metal surgical implants and metal levels in biological tissues has significantly grown.

In particular, in orthopaedics, this issue has been widely discussed and studied for many years. Wear and corrosion of metallic prosthetic components can result in ion release into surrounding tissues, particularly chromium and cobalt. Other materials, such as titanium, can also be released from such devices [10, 11].

This phenomenon can lead to severe local tissue reactions up to necrosis and potential systemic adverse effects due to dispersion into the bloodstream of ions. Peri-implant biological analyses can detect the presence of metals compatible with device composition and trace metals can also be found in tissues and organs far from the primary site [12, 13].

MIRPE technique was initially described by Nuss [14] and is nowadays considered the gold standard for pectus excavatum repair and its popularity is increasing. Treated patients are in most cases young, which enhances the need in determining possible local and systemic impact of implanted metallic devices in the short and long term.

Cundy and Kirby were the first to raise attention to trace metal levels in the blood of patients undergoing MIRPE in 2012 [15] and described an increased serum chromium.

Fortmann et al. [4] found elevated metal values in various biological tissues, including plasma, urine, and peri-prosthetic tissue.

In 2021 [3] we analysed blood metal levels in two groups of patients pre and post maintenance of bars and stabilizers and we found a significant increase in blood chromium values and a general upward trend in the metals studied. At the same time, stabilizers seemed to correlate to higher levels of blood of the metals, suggesting a release caused mainly by friction between the metal components.

In the present paper, we performed a longitudinal evaluation in the same patient before and after bar and stabilizer maintenance, thus reducing one of the major biases of most previous studies, namely individual variability.

We confirm that chromium concentrations increase in patients after MIRPE, both considering the percentage of patients above the threshold and considering the mean value pre- and post-surgical correction. Interestingly, differently from our previous findings, in this work we were not able to find any significant difference correlated to the number of stabilizers used. At least one stabilizer was always used in this population so it was not possible to perform analyses on patients without stabilizers to possibly confirm previous evidence.

The clinical impact of metal release in patients after MIRPE has not yet established. Systemic toxicity effects from metal release have never been reported in the PE population, to our knowledge. However, we cannot exclude potential general toxicity consequences in patients with such increased blood metal levels. From a clinical perspective, evidence of toxicity related to metal accumulation may become apparent only long after the initial exposure [16]. Consequently, the absence of symptoms during the three-year period of device retention does not allow us to exclude the possibility of late-onset complications in otherwise asymptomatic patients. Metals circulating in the bloodstream are known to undergo multi-organ bioaccumulation, and their toxic effects are typically slow and insidious, potentially remaining clinically silent for prolonged periods [17].

Evaluating the potential risk of metal toxicity in patients operated for MIRPE, we have to consider that abnormal metal levels can be found in different biological tissues and for a long time after MIRPE according to the studies of Fortmann. Also according to Seyrek and Akkus [16], the values of metals such as nickel have found to be increased even after devices removal, as demonstrated by studying hair samples.

In our opinion, the results of our study further enhance the need of investigating the role of metal release in PE population. This is paramount, considering that pectus excavatum population is mainly composed of young and otherwise healthy patients, who are exposed to unknown risks for a prolonged period.

Moreover, it has been hypothesized that local irritative symptoms, such as local swelling, seroma, wound healing disorders, or pleural effusion, can be associated with metal release after MIRPE. These signs must be differentiated from metal allergy which seems to appear in 0.5–6.4% of MIRPE patients [17]. Interestingly, preoperative metal allergy testing doesn’t seem to be associated with a reduction in bar allergy symptoms [18].

Fortmann et al. found an increased risk of irritative symptoms of the soft tissue and pleural effusion in patients with abnormal tissue metal levels. In contrast, none of the patients described in our study presented similar symptoms.

Solutions to lowering the risk of metal release such as, for example, a titanium nitride coating apparently do not display any protective effect if compared with uncoated device placement [19]. The use of titanium bars instead of steel bars could be another attempt of reducing toxicity by metal release; however we do not have any data to support this at the present time.

Differently from our previous study, in which different cohorts of patients were compared, in the present study we have analysed metal levels in the same cohort pre and post MIRPE, which represents the main point of strength. The number of patients, although higher than in other studies, remains the main limitation of this study as it cannot be excluded that results could be subjected to wider variations when considering a larger sample size. Another critical issue that should not be underestimated is the possible interference of external variables in the concentration of trace metals (e.g., diet, geographical area). Another limitation of this study is the use of reference values for the adult population.

## Conclusion

Patients undergoing MIRPE displayed increased blood levels of chromium and molybdenum. At present, there is no correlation between blood levels and symptoms, as observed, in the short- and medium-term follow-up. However, a pathological role of these results cannot be ruled out with certainty. Although generally considered safe from the point of view of biocompatibility, future developments of MIRPE also depend on the evolution of materials.

**Table 1:** Intrauma (Intrauma, Torino, Italy) stainless steel chemical composition for bars and stabilizers; ISO 5832-1:2016

Element	Mass fraction %
Carbon	0.030 max
Silicon	1.0 max
Manganese	2.0 max
Phosphorus	0.025 max
Sulfur	0.010 max
Nitrogen	0.10 max
Chromium	17.0 to 19.0 max
Molybdenum	2.25 to 3.00
Nickel	13.0 to 15.0
Copper	0.50 max
Iron	Balance

**Table 2:**
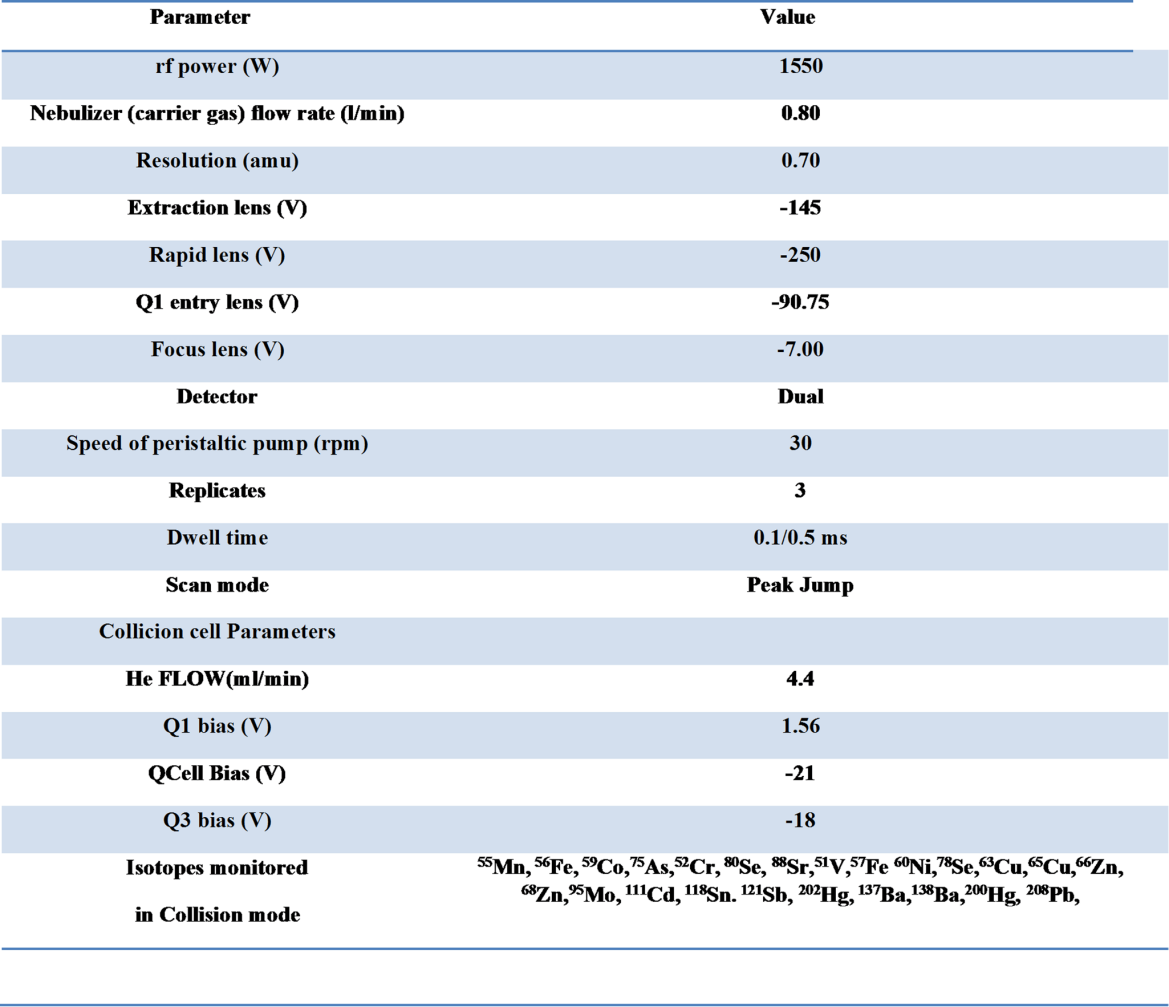
Parameters and operating conditions for the ICP-MS instrument

**Table 3:** Mean metal values pre and post MIRPE devices

Chromium	Patients	Pre MIRPE mean ± sd	Post MIRPE mean ± sd	*p* value
	53	0.52 ± 0.43	2.43 ± 9.57	< 0.001
Manganese	53	12.69 ± 7.84	19.81 ± 45.97	0.26
Molybdenum	53	0.35 ± 0.39	1.87 ± 1.29	**< 0.001**
Nickel	53	4.24 ± 12.50	80.80 ± 305.28	0.31

*MIRPE* Minimally Invasive Repair of Pectus Excavatum

**Table 4:** Metals values according to number of bars

	*N*° bars = 1 *N* = 33	*N*° bars > 1 *N* = 20	*p* value
	mean ± SD	mean ± SD	
Chromium PRE	0.56 ± 0.44	0.44 ± 0. 39	0.32
Chromium POST	3.33 ± 12.07	0.93 ± 1.09	0.38
Manganese PRE	12.05 ± 8	13.76 ± 7.64	0.44
Manganese POST	24.15 ± 58.09	12.65 ± 3.72	0.38
Molybdenum PRE	0.23 ± 0.35	0.54 ± 0.39	**0.001**
Molybdenum POST	1.92 ± 1.46	1.81 ± 1	0.77
Nickel PRE	3.06 ± 5.65	6.18 ± 19.17	0.38
Nickel POST	72.62 ± 231.24	94.30 ± 405.83	0.81

*MIRPE* Minimally Invasive Repair of Pectus Excavatum, *PRE* pre-operative (before devices implantation), *POST* post-operative (after devices removal), *SD* Standard Deviation

## Data Availability

The data underlying this article will be shared on reasonable request to the corresponding author.

## Abbreviations

MIRPE: Minimally Invasive Repair of Pectus Excavatum
PE: Pectus Excavatum
ICP-MS: Inductively Coupled Plasma – Mass Spectrometry
